# Supplementary material for: Impact of outer width of the metacarpal diaphysis on the identification of low bone mass in children
Source: PLoS One. 2025 Nov 7;20(11):e0336393. doi: 10.1371/journal.pone.0336393 (PMC12594424; doi:10.1371/journal.pone.0336393)
Supplement: Attachment — Submitted filename: Jepsen - Response to Reviewers.docx [file pone.0336393.s002.docx]

**PONE-D-25-37020**

**Identifying girls and boys with low bone mass from hand radiographs may be improved by considering the variation in metacarpal external size**

**Response:** We thank the Associate Editor and both reviewers for their enthusiasm of our paper and their constructive comments. We responded positively to all comments and feel the paper has been substantially improved. We look forward to the review of our revision and the opportunity to address any remaining questions.

=================================

**EDITOR**

**In addition to the Reviewers' comments, I note the following:**

1. Please ensure the authors comply with PLOS One's data availability policy - data should be submitted to a public repository.

***Response:*** The measured and calculated metacarpal data used in this study are being uploaded to a publicly accessible resource. Please note that this data upload has just been initiated and will be complete within a few days. The doi for this data is: https://doi.org/10.7302/47ej-xb63

Access to the hand radiographs is publicly available but only through Bolton-Brush Growth Study Center at Case Western Reserve University; <https://case.edu/dental/departments-programs/bolton-brush-growth-study-center>

2. While I appreciate the authors' expertise and previous work in this area, I find it somewhat disappointing that the authors are recommending development of yet another diagnostic tool for bone mass assessment. The bone research field has benefited from the introduction of tools such as pQCT & HR-pQCT, yet clinicians still rely mostly on DXA and this is the likely to remain the case. Is there a way for what is learned in this proof of concept study to be applied to other existing imaging tools beyond hand radiographs?

***Response:*** We would like to address the Associate Editor's comments with two points. First, we do not feel the current paper is recommending 'yet another' diagnostic tool for bone mass assessment; in fact, we are proposing a refinement of existing assessments to reduce bias within populations which is an area of research I believe many would feel is critically important. Further, the X-ray technology used to generate hand radiographs is more prevalent and thus more accessible than pQCT, HRpQCT, and DXA, which are expensive; as such, beginning this analysis using hand radiographs is highly appropriate for democratizing access to a diagnostic tool that minimizes bias. Second, we reframed the focus of the study to be less about advocating for use of hand radiographs compared to other systems to one where we are advocating adjustments for measures like external size which may be a source of bias. How the external size dependent differences in bone mass are observable in the various technologies (pQCT, HRpQCT, DXA) has yet to be determined. Given the results of this study, we would recommend that additional research is needed to study sources of bias in diagnosing low bone mass such as that reported herein.

***Changes in the revision:*** As noted in the response to reviewer 1, we removed the section of the Discussion (page 9) that compares the advantages and limitations of different imaging technologies.

3. A couple of minor formatting points: First, males/females is more appropriate when referring to biological sex, whereas boys/girls is used to refer to gender roles. Second, the abbreviation for cortical area can be used throughout the manuscript after it is first defined (which could be in the 2nd paragraph of the introduction on page 3). There are several places in the manuscript (other than at the start of a sentence) where cortical area is spelled out in full, but the abbreviation could be used instead (i.e., in the last sentence of the intro).

***Response:*** We replaced girls/boys with females/males throughout the paper and used Ct.Ar and Tt.Ar abbreviations after the first call out.

4. Abstract, 4th sentence: Consider adding a definition for total area as was done for cortical area in this sentence. Currently, someone could read this as total and cortical area being measures of bone mass.

***Response:*** This sentence was modified as follows: ***"****Total area (Tt.Ar), a measure of the area enclosed by the periosteal surface, and cortical area (Ct.Ar), a measure of bone mass, were calculated assuming a circular cross-section."*

5. Sample population: Does the current dataset represent all ~8 year olds in the Broadbent-Bolton Collection? Did the authors have to exclude any radiographs from the analysis. Also, at the top of page 5, the authors mention "the power needed to test the current hypothesis" but no information on a power / sample size calculation was provided in the manuscript.

***Response:*** We updated the manuscript to indicate that the radiographs were obtained for a random sampling of children enrolled in the Bolton-Brush study. page 4, *"The hand radiographs were purchased from the Broadbent-Bolton Collection at the Bolton-Brush Growth Study Center in 2007 and represent a randomly selected subgroup of those enrolled in this study."*

We corrected the missing power analysis by including the following text in the Methods section, page 5: *"The combined dataset allowed us to test for an effect size as low as 0.45 when comparing the tertiles (3 groups) with 80% power and a significance level of 0.05. This study was powered to test for significant differences in Ct.Ar among tertiles, which showed an average effect size of 1 for females and males."*

6. Bone structural measures: Please provide the units of measurement in the text. Please provide a reference for the statement regarding replacement of slenderness with robustness.

***Response:*** We removed this sentence to reduce confusion as the prior two papers reporting bone morphology from the Bolton-Brush used the term 'robustness' rather than slenderness. Our inclusion of this sentence refers to other papers that are not germane to the current study and so there was no need for this clarification.

7. Statistics: It would help if the authors could clarify how the Z-score thresholds were varied.

***Response:*** We added the following text to the Results section (page 8) to clarify how the Z-score thresholds were varied: *"For females, the Z-score thresholds were -0.51 and -0.40 so 33% of individuals were identified with low bone mass when compared to the group average and tertile-specific average, respectively. For males, the Z-score thresholds were -0.70 and -0.36 so 33% of individuals were identified with low bone mass when compared to the group average and tertile-specific average, respectively."*

8. Results: Relative cortical area is mentioned in the first paragraph, but this outcome wasn't defined in the methods. Similarly, BMI is presented in the tables but is not mentioned in the text.

***Response:*** We added new text to the Methods section to define BMI *(page 5: "Body Mass Index (BMI) was calculated as body weight divided by height squared.")* and relative cortical area *(page 6: "Relative cortical area (RCA) was calculated as Ct.Ar / Tt.Ar.")*

=========================================

**Reviewer #1:** Thank you for the opportunity to review this manuscript. The research has been undertaken with rigour and adds to our understanding of bone structure in children. However, the manuscript would benefit from minor revision. My edits and comments are presented below.

***Response:*** We thank the reviewer for the kind words and constructive comments.

**1. Title: The title should be modified to sound less like a conclusion and more like a title.**

***Response:*** The new title reads, "Impact of external size on the identification of low bone mass in children"

**2. Abstract**

**• Include whether left or right hand radiograph was used**

***Response:*** Hand radiographs were obtained for the nondominant hand. This designation is now included in	the Abstract (as suggested) and in the Methods (page 4).

**3. Lay summary**

• The first sentence refers to findings from a previous study – perhaps rephrase this sentence so that it reads as “background”.

***Response:*** The first sentence now reads, *"In prior work, we reported that bone mass varied with outer bone	size."*

• The second sentence should be rephrased for clarity “We showed in this study that this association…”

***Response:*** The second sentence now reads, *"In the current study, we showed that this association affected	the	identification of female and male children with low metacarpal cortical area."*

**4. Introduction**

• Line 4 – “The current diagnostic approach...” It is unclear what this phrase refers to.

***Response:*** The sentence was revised to provide clarity regarding the diagnostic approach we are referring to: *"The current approach to assessing bone strength is tailored for rare diseases and related conditions but is neither beneficial for the general population nor effective in establishing comparative norms."*

• Paragraph 2 - A radiograph is the image obtained from x-ray exposure. Therefore, the term “radiograph” should be used instead of “x-ray” when referring to the image. This should be corrected throughout the manuscript.

***Response:*** Thank you for pointing this out. We replaced X-ray with radiograph throughout the manuscript,	except when used in the titles of cited papers.

• Paragraph 2 - A reference is required for “Another potential source of bias which may not be immediately obvious beyond sex and race/ethnicity is the variation in bone structure itself”

***Response:*** We cited our prior study examining how external bone size affects the association between metacarpal structure and strength.

E. M. R. Bigelow, R. W. Goulet, A. Ciarelli, S. H. Schlecht, D. H. Kohn, T. L. Bredbenner, et al., Sex and external size specific limitations in assessing bone health from adult hand radiographs, *JBMR Plus* 2022 Vol. 6 Issue 8 Pages e10653

**5. Materials and Methods**

Sample population and data collection

• Page 4 The section below belongs in the Introduction of the manuscript where the background, rationale and objectives of the current study should be presented.

“Further, the current study builds on our prior work which established different external size dependent longitudinal trajectories for bone traits during growth (23, 24). Our prior work showed that the morphological traits of narrow bones grow along a different trajectory compared to wide. The current paper builds on our prior work by clarifying the differences in Ct.Ar among the narrow, intermediate, and wide subgroups, and then goes a step further to test if assessing low Ct.Ar can be improved by taking the variation in external size into consideration”

***Response:*** Thank you for this suggestion. We moved this text to the Introduction (page 4), which now connects our overall premise to our prior longitudinal study more clearly.

• “Our prior work showed that the morphological traits of narrow bones grow along a different trajectory compared to wide” – A reference is required

***Response:*** Our prior two studies (Pandey et al, JBMR 2009; Bhola et al, Bone 2013) are now included in this sentence.

• “The current paper” should read “the current study”

***Response:*** The 'current paper' was replaced with 'current study.'

• Specify whether the radiographs of the left or right hand

***Response:*** Hand radiographs were taken of the nondominant hand. This is now included in the Abstract and Methods sections.

**6. Bone Structural Measures**

• Whilst the methodology has previously been published, details on the tools used to perform measurements should be included in this manuscript

***Response:*** There were not many more details to add regarding the measurement protocols except to clarify that the measures were conducted manually using the software and that the coefficient of variation for repeat point to point measures was 1.61%. This information is now included in the revised Methods section (page 5).

• Page 5 – “Given that bigger people tend to have bigger bones” – specify what “bigger” means, and avoid the use of lay terms.

***Response:*** The reviewer asks a good question and our apologies for the laid-back terminology. We removed this parenthetical phrase to avoid using lay terms and to just state that Tt.Ar was adjusted for bone length to take the inter-individual variation in body size into consideration.

**7. Analytical Methods**

• Paragraph 1 – The following sentence should be rephrased or fragmented for clarity - “The primary hypothesis is that consideration of external size-dependent differences in bone structure will affect the identification of individuals with lower Ct.Ar compared to the standard method, which compares individuals to a group average.

***Response:*** The sentence was rewritten as follows: *"We tested two hypotheses in the current study. First, we tested whether Ct.Ar differed among subgroups sorted based on external bone size. Second, we tested whether identifying individuals with lower bone mass would be improved when comparing Ct.Ar of individuals to the average of their external size subgroups versus comparing Ct.Ar of individuals to the overall cohort average."*

**8. Discussion**

General comments

Given that radiographs of only White children were assessed, the manuscript would benefit from mentioning ethnic differences in metacarpal bone structure.

***Response:*** The paragraph describing the limitations of the paper (page 11) includes a statement regarding the need to conduct a similar study in other databases having more diverse representation of children based on racial, ethnic, and ambulatory backgrounds.

*"This study has several limitations to address for a more comprehensive understanding of bone health assessment. First, the Bolton-Brush collection is limited to a specific demographic, focusing exclusively on ambulatory White girls and boys. This narrow demographic restricts the generalizability of our findings. Future research should include children from a wider range of backgrounds to understand how variation in bone structure affects low bone mass diagnoses across different racial/ethnic and ambulatory groups."*

**9. Specific comments**

• Page 8 Paragraph 1 – “The primary outcome measure was metacarpal cortical area, which provides a dual measure of bone mass and strength…..” The metacarpal cortical area is not a direct measure of bone strength as stated, but is a proxy for bone strength.

***Response:*** We rephrased this statement to read - "*The primary outcome measure was metacarpal cortical area, which provides a measure of bone mass and a proxy for whole bone strength that lacks a sex-specific bias (21)."*

• Page 9 – “Hand radiographs offer some advantages over conventional DXA methods by providing a more detailed view of bone structure….” Please clarify the highlighted phrase – what additional details do you get from a radiograph compared to DXA? Additionally, Reference 37 that has been used to support this sentence has been misinterpreted.

***Response:*** We removed this section of the Discussion as a comparison of the advantages and limitations of different imaging technologies is not germane to the study outcomes.

• Page 10 Paragraph 2 “Although narrower bones have a lower baseline strength (24, 40), there are some advantages to having this structural phenotype. Recent research indicates that women and men with narrower femoral necks….” Firstly, whilst the authors state that there are “some” advantages, only one is cited. Are there others? Secondly, given that later in the manuscript the authors mention the inability to generalize their results to other sites, it is odd that they have cited a study on the adult femoral neck to substantiate their comment.

***Response:*** There may be other advantages but we only cite the one; as such the sentence was rewritten as follows: *"Although narrower bones have a lower baseline strength (24, 37), there is at least one advantage to having this structural phenotype."*

The inconsistency noted by the reviewer regarding whether the outcomes of our study of the metacarpal would be observed for other long bones is addressed in your next question.

• Page 12 paragraph 1 - “Associations between the development of the metacarpal and the development of other long bones was not investigated in this study. Consequently, we do not know if the results of the current study are generalizable to other anatomical sites”. Whilst the authors do not know whether their metacarpal results are applicable to other sites, perhaps they can discuss based on the known differences between metacarpals and other long bones, and from the results of previous studies that have made this comparison.

***Response:*** We reframed these statements to include evidence that Ct.Ar does indeed vary among long bone diaphyses for adults and that bone mineral content varies with external size of the proximal femur for older women and men. We do not know whether the association between these measures of the amount of bone and external bone size affects the identification of individuals with low bone mass. This is an active area of investigation by Jepsen and Bredbenner that we hope to resolve in the next few years. We replaced the sentences noted above with the following text (page 12): *"Although cortical area has been shown to vary with external size for other long bone diaphyses of adult females and males (21, 45) and bone mineral content has been shown to vary with the external size of the proximal femur of older females and males (38), additional studies are needed to confirm whether the association between the amount of bone and external bone size affects the identification of individuals with low bone mass at other anatomical sites."*

• Page 12 “In conclusion, this study provides evidence that the variation in bone structure arising from the complex adaptive nature of bone may be a source of bias when identifying individuals with low bone mass.” This study has not investigated any factors that influence bone size/structure. Therefore, I fail to understand the inclusion of the highlighted phrase.

***Response:*** Thank you for pointing this out. This sentence was rephrased to be more specific to the study outcomes and to remove the suggestion that we studied factors influencing bone size. The sentence now reads: *"In conclusion, this study provided evidence that the variation in Ct.Ar relative to the external bone size of the metacarpal may be a source of bias when identifying individuals with low bone mass."*

**Reviewer #2:** Thank you for the opportunity to read and review this manuscript, “Identifying girls and boys with low bone mass from hand radiographs may be improved by considering the variation in metacarpal external size”. This research uses hand-wrist radiographs from a longitudinal study of known sex and age individuals to evaluate the impact that metacarpal robusticity has on bone mass. I found this manuscript interesting and well-written; it has a number of strengths, but there are a few areas (detailed in no particular order below) that may require some additional attention.

***Response:*** We thank the reviewer for their kind words regarding the manuscript and the constructive nature of the suggested edits.

Some thoughts that I had while I was reading this manuscript:

I found the paper aims to be really interesting and insightful. While we frequently control for body size in our metacarpal analyses, this is often done to address sex-related size discrepancies. Based on the results of this paper, it seems clear that the bone robusticity (narrowness) does impact the cortical area, even after the element’s length is controlled for.

Perhaps my biggest question is in regards to the method and measurement justification. Specifically, the authors use the elliptical model method (EMM) to mathematically estimate the metacarpal cortical area from a single-plane radiograph. Typically, with a single plane X-ray (rather than a CT), I may choose a technique such as metacarpal radiogrammetry to measure the cortical thickness, which could then be corrected by the total width (thereby accounting for the bone’s robusticity/narrowness). Although it is a much simpler approach, metacarpal radiogrammetry has numerous clinical comparators, has been shown to correlate with bone mass, and does not mathematically extrapolate bone properties (as is necessary with EMM). While I do not disagree with the use of cortical area (there are many great reasons why it might be necessary!), I was curious: in this research, why is cortical area the necessary analytical parameter? Is this the standard in children’s bone mass analyses? Or is it because cortical area is specifically linked with a biomechanical attribute? I do not think it would be a large addition, but I would like to see the authors acknowledge/explain their use of the EMM over the more conventional metacarpal radiogrammetric approaches (i.e., why EMM / why not radiogrammetry).

***Response:*** The reviewer raises an interesting point. We used Ct.Ar measured directly from the radiographs based on a prior study we conducted comparing various morphological parameters to whole bone strength {Bigelow, 2022 #2065}. This study used adult human cadaveric tissue so we had direct measures of whole bone strength. Our goal was to identify those parameters that did not show a sex specific difference between the morphological trait and whole bone strength. Cortical area was the only morphological parameter to not show a statistically significant difference in the association with whole bone strength. We also used Ct.Ar because it is a direct measure of the amount of bone (in a cross-sectional plane) and thus also serves as a measure of bone mass for the metacarpal. The parameter the reviewer refers to - cortical thickness normalized by total width, also known as the metacarpal index (MCI) - showed a significantly different association with whole bone strength between females and males. Admittedly, we were hoping MCI would not show a sex-specific difference since this parameter is nondimensional and would then allow us to assess a proxy of strength from any radiograph, even those without a calibration marker. Unfortunately, this was not the case. This explanation may explain in part why we did not use radiogrammetry in this study to assess bone mass.

We added a new sentence to the Methods section (page 6) to address the point raised by the reviewer:

*"Ct.Ar was used as a measure of bone mass rather than other parameters used in radiogrammetry such as the metacarpal index (MCI = cortical thickness normalized by total width) {Oza, 2023 #2111}, because the association between parameters like MCI and whole bone strength showed a significant difference between females and males {Bigelow, 2022 #2065} and our goal was to minimize bias."*

I found Figure 2 quite visually effective. Nice addition.

***Response:*** We very much appreciate the comment.

Statistical reporting: I would encourage the authors to report their statistics in full, including the test result and degrees of freedom (when applicable), in addition to the p-value.

***Response:*** We included additional statistical read outs where it seemed appropriate, for example the ANOVA tests (Table 2; now include the F-statistic, degrees of freedom, and p-values) and the chi-square tests (Table 3 now includes the degrees of freedom, sample size, and chi-square statistic).

I found myself getting a little turned around with the difference between tertile and robustness tertile in the results section and in the figures/tables. For example, I understood that the robustness categories were also defined by tertiles – so interpreting the differences between Fig 3 B & C was a little difficult initially. I presume C is the randomized tertile? In text, for example, pg 7 para 2: “Relative cortical area differed among the tertiles for males but not for females.” – does this mean it differed for both randomized AND for robustness tertiles? Or just randomized? I would find a way to refer to these tertile groups in distinct ways in the text, tables, and figures. Perhaps once you introduce them as having been generated as tertiles, they might be called robustness tertiles vs. randomized tertiles? And these more descriptive categorical labels should be applied to the figures as well.

***Response:*** Thank you for this suggestion and we agree with the new labeling scheme. The revision now	refers to the tertiles as robustness tertiles and randomized tertiles throughout the manuscript.

Pg 9, para 2 – this paragraph was really nicely discussed – I found it particularly helpful in better understanding the narrow vs. wide strength argument in relation to the author’s findings. Nice.

***Response:*** We very much appreciate the comment.

How would the authors propose these findings be applied in a clinical setting, in the absence of population standards? Based on these results, can a very general guideline be proposed to identify what might be considered low bone amounts as they relate to different metacarpal morphologies? If not, how should this work move forward so that children’s bone mass is being better quantified and categorized according to morphology?

***Response:*** The reviewer raises a good question and one that could certainly be made more intentional in the paper. The revision includes the following new text in the conclusion paragraph (page 13): *"The results of this study suggest that additional research is needed to identify sources of bias that may affect the identification of individuals with low bone mass. This research could be impactful clinically if new approaches can be developed to refine existing diagnostic technologies (e.g., radiogrammetry, DXA, HRpQCT)."*

Other minor edits:

- Pg 4, para 1: “For the metacarpal, cortical area (Ct.Ar) is a direct measure of the amount of bone (i.e., bone mass) for THE diaphysis…” (missing a word, could also perhaps read “in the diaphysis”, or “of the diaphysis”.

***Response:*** Thank you for catching this. The sentence was corrected to include "the".

Overall, this manuscript offers valuable insight into factors, such as robusticity, that impact the ability to accurately assess and interpret low bone mass in metacarpals. I believe the findings reported in this study are important, and understanding the role of diaphyseal robusticity in quantifying cortical bone mass will be highly valuable for future assessments, potentially even in adults.

***Response:*** We very much appreciate the comment.

________________________________________
