# Supplementary material for: Impact of outer width of the metacarpal diaphysis on the identification of low bone mass in children
Source: PLoS One. 2025 Nov 7;20(11):e0336393. doi: 10.1371/journal.pone.0336393 (PMC12594424; doi:10.1371/journal.pone.0336393)
Supplement: Attachment — Submitted filename: Jepsen - Response to Reviewers 2nd revision.docx [file pone.0336393.s004.docx]

**Comments to the Author**

**Reviewer #1:** Impact of external size on the identification of low bone mass in children

The reviewer thanks the authors for addressing the comments. Whilst mostly this has done been satisfactorily, there are still points which require clarification.

**Title**
Whilst the revised title now reads less like a conclusion, it still needs improvement! What does “external size” refer to? Similarly, the revised sentence on p5 needs to clarify ‘external size”

***Response:*** External size was replaced with "outer width of the metacarpal diaphysis" in the title. External bone size was defined in the Abstract (page 2) and when it was first used in the Introduction (page 3).

New text (page 2; Abstract):  *"Prior work showed that bone mass varied with external bone size, a measure of the outer bone width."*

New text (page 4; Introduction):  *"External bone size represents any measure describing the outer size of the bone, including outer width and total cross-sectional area."*

**Abstract**
The issue of hand dominance remains unclear.
The authors were asked to specify whether left or right hand radiographs were used. The authors instead now mention use of the radiograph of the non-dominant hand. Does this mean that there was a record of whether an individual was sinistral or dextral, and that a combination of left and right hand radiographs was used? Or did they assume that all individuals were right-hand dominant?
Kindly address this in Methods as well

***Response:*** We would assume the study records included an assessment of hand dominance, but we did not have access to this information. The majority of the radiographs examined were of the left hand, which is consistent with the statistic that approximately 90% of individuals are right hand dominant.

New text (page 5, Methods): *"The majority of the radiographs examined were of the left hand, which is consistent with the general statistic that approximately 90% of individuals are right hand dominant."*

**Introduction**
Page 3 - “A review of morphological measures derived from hand radiographs (20) revealed that many widely used measures correlated with experimentally determined strength but with a sex-specific discrepancy (21).” – reference 20 is inappropriately placed. Are both refs 20 and 21 required? Citing an empirical study rather than a review is preferred.

***Response:*** We removed the reference to the review paper and only used the empirical study when referring to prior work showing a sex-specific discrepancy between morphological measures and bone strength.

Page 4 - “Narrower bones have proportionally thicker cortices but lower cortical area, Ct.Ar (i.e., mass) compared to wider bones, but adjustments for this morphological variation have not been considered clinically or for research.” – the highlighted portion is inaccurate. I suggest the authors review the longitudinal studies on metacarpal bones undertaken in South African children.

***Response:*** We thank the reviewer for pointing out this error in referencing prior work. We included two papers reporting on longitudinal bone changes in South African children as examples of studies which took into consideration the associations between external bone size and the amount of bone.

New text (page 4, Introduction): *"Narrower bones have proportionally thicker cortices but lower Ct.Ar (i.e., mass) compared to wider bones {Magan, 2017 #2112;Magan, 2019 #1956}."*

Refs 22 and 23 were studies done on the same data set as the current study i.e. white individuals. Therefore, I fail to see how these 2 refs were used to cite racial/ethnic differences in bone.

***Response:*** Thank you for pointing this out. Although the references were intended to point out the range in external sizes and not work conducted on multiple races/ethnicities, we see now how this was an error. We adjusted the text to remove the reference to races/ethnicities and to simply reference prior work examining the variation in external bone size independent of body stature.

New text (page 4; Introduction): *"External bone size varies within female and male populations, ranging from narrow to wide phenotypes, even after adjusting for body stature (21, 22)."*

**Methods**
Page 6. “Body Mass Index (BMI) was calculated as body weight divided by height squared.” – please provide units of measurement.

***Response:*** Units are now included in the revised manuscript.

New text (page 6; Methods): *"Body Mass Index (BMI) was calculated as body weight (kilograms) divided by height squared (meters^2^).”*

Were the radiographs digitised or hard copies? In the context of my question, please clarify how measurements were then done “manually”.

***Response:*** The measurements were determined from digitized copies of the original radiographs. The text was updated to reflect this detail. Measurements were done manually in the sense that the student identified the edges of the outer and inner surfaces of the diaphysis and did not use an edge detection algorithm.

New text (page 5, Methods): *"Digitized hand radiographs were purchased from the Broadbent-Bolton Collection at the Bolton-Brush Growth Study Center in 2007 and represent a randomly selected subgroup of those enrolled in this study."*

New text (page 6, Methods): *"Manual point-to-point measurements refer to the identification of the edges of the outer and inner surfaces of the diaphysis by an individual and not by an edge detection algorithm."*

“Manual point-to-point measurements of outer and inner bone widths had an average coefficient of variation of 1.61%.” This appears to be a citation from ref 23 where data for only 29 boys and girls were used. Given the differences in sample sizes, it seems coincidental that the coefficient of variation in this study is identical to that of the previous study (ref 23)? Kindly explain.

***Response:*** The repeatability study was conducted at the time the measurements were taken using a random collection of hand radiographs (n=5) with 10 repeat measurements each for the outer and inner widths. The coefficient of variation was averaged for the outer and inner width measures. The Methods section was updated to include this information.

New text (page 6, Methods): *"A repeatability study was conducted at the time the measurements were taken using a random collection of five hand radiographs with 10 repeat measurements for each of the outer and inner widths."*

**Reviewer #2:** Thank you for the opportunity to read and review the revised version of this manuscript, “Impact of external size on the identification of low bone mass in children”. This research uses hand-wrist radiographs from a longitudinal study of known sex and age individuals to evaluate the impact that metacarpal robusticity has on bone mass. I thought the authors did a good job of integrating and applying the reviewer feedback. I am happy with the revisions, but they have raised a few additional comments (detailed in no particular order below) that may require some additional attention.

Some thoughts that I had while I was reading this manuscript:

In the response to reviewers, the authors reiterate how important it was that they could pool sexes for these analyses. Initially, I found this a little unclear as to why – especially because on pg 4 the authors state the reference groups are often split by sex and age, and the sex differences mentioned for MCI measures made me worry that sex was indeed a confounding factor that should be controlled for. *However*, nearer the end of the paper, the authors bring up an excellent point about how a non-sex specific approach is more inclusive in cases of sex- and gender-diversity. This is an excellent point, and one that could perhaps be emphasized in the earlier justifications for why this study aimed to derive the sample they did (i.e., both sexes pooled).
***Response:*** We apologize for the confusion. The male and female data were not pooled but analyzed separately. We reviewed the Methods section and made some tweaks to the text to ensure this point was clear.

Revised text (page 6, Methods): *"The female and male datasets allowed us to test for an effect size as low as 0.45 when comparing the tertiles (3 groups) with 80% power and a significance level of 0.05." "This study was powered to test for significant differences in Ct.Ar among tertiles, which showed an average effect size of 1 for females and males when analyzed separately."*

Revised text (page 7, Analytical Methods): *"We tested two hypotheses in the current study with females and males being analyzed separately."*

*Revised text (*page *10, Discussion): "Hand radiographs from eight-year-old females and males were examined and analyzed separately as a proof-of-concept to test whether metacarpal external size affected the identification of children with lower Ct.Ar."*

Pg 7, Results para 1: An ANOVA p-value is reported without the associated F and df. These are likely in the table, but if the p-value is presented in text, perhaps the authors could also consider in-text presentation?
***Response:*** The Results section now includes ANOVA details for body weight, height, and metacarpal length.

Revised text (page 9, Results): *"Weight (Females: F(2)=0.16, p=0.849; Males: F(2)=1.00, p=0.376; ANOVA), height (Females: F(2)=1.51, p=0.234; Males: F(2)=0.36, p=0.698; ANOVA), and metacarpal length (Females: F(2)=0.40, p=0.673; Males: F(2)=0.002, p=0.998; ANOVA) did not differ among the robustness tertiles for either sex (Table 2), indicating that sorting females and males based on second metacarpal robustness reflected differences in outer bone size but not body stature or bone length."*

Statistics presentation: I would encourage the authors to adhere to other, more common or accepted guidelines for how to present df and F statistics alongside p-values. For ANOVA, this may look like (a completely fictional example): (F(2)=9.073, p=.031). The ‘2’ in brackets after the F is the df. This reporting makes for a cleaner/tidier presentation of the stats in a way that matches convention in many studies. I would also encourage the tabulated statistics to be presented the same.
***Response:*** The revision now includes the recommended reporting format for ANOVA throughout the Results section and Table 2.

The final concluding sentence: I understand this is a new sentence addition, but I am not sure that it helps to clearly summarize your paper, or its findings and implications. Specifically, I am not convinced that we actually need new approaches, or that this paper supports that need specifically. We might, however, need measured ranges that can advise clinicians when to further investigate an individual as ‘at risk’ for low bone mass; I understand that these absolute ranges are not something that the authors can provide within the scope of this study. Perhaps instead, these final sentences may reiterate that the study did not endeavor to provide absolute narrowness/wideness values/ranges of concern for low bone mass, but rather to establish that bone width can misdirect, and therefore cannot be accurately used to identify loss conclusively. Based on the results of this research, future studies and clinical practice should be aware that even wide bones may still have strength and mass concerns/issues.

***Response:*** The concluding paragraph was modified, including removing the last sentence.

Revised text (page 14/15, Discussion): *"Whereas this study did not attempt to establish absolute ranges for outer bone size sub-phenotyping, the data do suggest that recognizing patterns of structural variation that exist among individuals and that arise from the complex adaptive nature of bone may improve our ability to identify individuals developing a suboptimal skeleton. Efforts aimed at refining diagnostic measures may benefit from additional research identifying sources of bias that affect the identification of individuals with low bone mass."*
