# Supplementary material for: Impact of outer width of the metacarpal diaphysis on the identification of low bone mass in children
Source: PLoS One. 2025 Nov 7;20(11):e0336393. doi: 10.1371/journal.pone.0336393 (PMC12594424; doi:10.1371/journal.pone.0336393)
Supplement: Attachment — Submitted filename: PONE-D-25-37020 Reviewer Comments.pdf [file pone.0336393.s001.pdf]

Identifying girls and boys with low bone mass from hand radiographs may be improved by considering the variation in metacarpal external size

Thank you for the opportunity to review this manuscript. The research has been undertaken with rigour and adds to our understanding of bone structure in children. However, the manuscript would benefit from minor revision. My edits and comments are presented below.

Title

The title should be modified to sound less like a conclusion and more like a title.

Abstract

- Include whether left or right hand radiograph was used

Lay summary

- The first sentence refers to findings from a previous study – perhaps rephrase this sentence so that it reads as "background".
- The second sentence should be rephrased for clarity "We showed in this study that this association…"

Introduction

- Line 4 – "The current diagnostic approach…" It is unclear what this phrase refers to.
- Paragraph 2 - A radiograph is the image obtained from x-ray exposure. Therefore, the term "radiograph" should be used instead of "x-ray" when referring to the image. This should be corrected throughout the manuscript.
- Paragraph 2 - A reference is required for "Another potential source of bias which may not be immediately obvious beyond sex and race/ethnicity is the variation in bone structure itself"

Materials and Methods

Sample population and data collection

- Page 4 The section below belongs in the Introduction of the manuscript where the background, rationale and objectives of the current study should be presented.

  "Further, the current study builds on our prior work which established different external size dependent longitudinal trajectories for bone traits during growth (23, 24). Our prior work showed that the morphological traits of narrow bones grow along a different trajectory compared to wide. The current paper builds on our prior work by clarifying the differences in Ct.Ar among the narrow, intermediate, and wide subgroups, and then goes a step further to test if assessing low Ct.Ar can be improved by taking the variation in external size into consideration"

- “Our prior work showed that the morphological traits of narrow bones grow along a different trajectory compared to wide” – A reference is required

- “The current paper” should read “the current study”

- Specify whether the radiographs of the left or right hand

Bone Structural Measures

- Whilst the methodology has previously been published, details on the tools used to perform measurements should be included in this manuscript
- Page 5 – “Given that bigger people tend to have bigger bones” – specify what “bigger” means, and avoid the use of lay terms.

Analytical Methods

- Paragraph 1 – The following sentence should be rephrased or fragmented for clarity - “The primary hypothesis is that consideration of external size-dependent differences in bone structure will affect the identification of individuals with lower Ct.Ar compared to the standard method, which compares individuals to a group average.

Discussion

General comments

Given that radiographs of only White children were assessed, the manuscript would benefit from mentioning ethnic differences in metacarpal bone structure.

Specific comments

- Page 8 Paragraph 1 – “The primary outcome measure was metacarpal cortical area, which provides a dual measure of bone mass and strength.....” The metacarpal cortical area is not a direct measure of bone strength as stated, but is a proxy for bone strength.

- Page 9 – “Hand radiographs offer some advantages over conventional DXA methods by providing a more detailed view of bone structure....” Please clarify the highlighted phrase – what additional details do you get from a radiograph compared to DXA? Additionally, Reference 37 that has been used to support this sentence has been misinterpreted.

- Page 10 Paragraph 2 "Although narrower bones have a lower baseline strength (24, 40), there are some advantages to having this structural phenotype. Recent research indicates that women and men with narrower femoral necks…." Firstly, whilst the authors state that there are "some" advantages, only one is cited. Are there others? Secondly, given that later in the manuscript the authors mention the inability to generalize their results to other sites, it is odd that they have cited a study on the adult femoral neck to substantiate their comment.

- Page 12 paragraph 1 - "Associations between the development of the metacarpal and the development of other long bones was not investigated in this study. Consequently, we do not know if the results of the current study are generalizable to other anatomical sites". Whilst the authors do not know whether their metacarpal results are applicable to other sites, perhaps they can discuss based on the known differences between metacarpals and other long bones, and from the results of previous studies that have made this comparison.

- Page 12 "In conclusion, this study provides evidence that the variation in bone structure arising from the complex adaptive nature of bone may be a source of bias when identifying individuals with low bone mass." This study has not investigated any factors that influence bone size/structure. Therefore, I fail to understand the inclusion of the highlighted phrase.
