# Supplementary material for: Impact of outer width of the metacarpal diaphysis on the identification of low bone mass in children
Source: PLoS One. 2025 Nov 7;20(11):e0336393. doi: 10.1371/journal.pone.0336393 (PMC12594424; doi:10.1371/journal.pone.0336393)
Supplement: Attachment — Submitted filename: PONE-D-25-37020R1 Reviewer Comments Revised version.pdf [file pone.0336393.s003.pdf]

Impact of external size on the identification of low bone mass in children

The reviewer thanks the authors for addressing the comments. Whilst mostly this has done been satisfactorily, there are still points which require clarification.

Title

Whilst the revised title now reads less like a conclusion, it still needs improvement! What does "external size" refer to?

Similarly, the revised sentence on p5 needs to clarify 'external size"

Abstract

The issue of hand dominance remains unclear.

The authors were asked to specify whether left or right hand radiographs were used. The authors instead now mention use of the radiograph of the non-dominant hand. Does this mean that there was a record of whether an individual was sinistral or dextral, and that a combination of left and right hand radiographs was used? Or did they assume that all individuals were right-hand dominant?

Kindly address this in Methods as well

Introduction

Page 3 - "A review of morphological measures derived from hand radiographs (20) revealed that many widely used measures correlated with experimentally determined strength but with a sex-specific discrepancy (21)." – reference 20 is inappropriately placed. Are both refs 20 and 21 required? Citing an empirical study rather than a review is preferred.

Page 4 - "Narrower bones have proportionally thicker cortices but lower cortical areaCt.Ar (i.e., mass) compared to wider bones, but adjustments for this morphological variation have not been considered clinically or for research." – the highlighted portion is inaccurate. I suggest the authors review the longitudinal studies on metacarpal bones undertaken in South African children.

Refs 22 and 23 were studies done on the same data set as the current study i.e. white individuals. Therefore, I fail to see how these 2 refs were used to cite racial/ethnic differences in bone.

Methods

Page 6

"Body Mass Index (BMI) was calculated as body weight divided by height squared." – please provide units of measurement.

Were the radiographs digitised or hard copies? In the context of my question, please clarify how measurements were then done “manually”.

“Manual point-to-point measurements of outer and inner bone widths had an average coefficient of variation of 1.61%.” This appears to be a citation from ref 23 where data for only 29 boys and girls were used. Given the differences in sample sizes, it seems coincidental that the coefficient of variation in this study is identical to that of the previous study (ref 23)? Kindly explain.
